# Method to measure the size of a radiographic field larger than a detector by imaging fluorescence X‐rays with a slit camera

**DOI:** 10.1002/acm2.13426

**Published:** 2021-09-23

**Authors:** Andreu Badal

**Affiliations:** ^1^ Division of Imaging, Diagnostics, and Software Reliability Office of Science and Engineering Laboratories Center for Devices and Radiological Health U.S. Food and Drug Administration Silver Spring Maryland USA

**Keywords:** collimator, fluorescence, mammography, quality control, radiography, slit camera, X‐ray field

## Abstract

**Purpose:**

X‐ray imaging devices contain a collimator system that defines a rectangular irradiation field on the detector plane. The size and position of the X‐ray field, and its congruence with the corresponding light field, must be regularly tested for quality control. We propose a new method to estimate how far the x‐ray field extends beyond the detector which does not require the use of external detectors.

**Methods:**

A metallic foil is inserted perpendicularly between the source and the detector. A slit camera, a linear extension of a pinhole camera, is used to project onto the detector the fluorescence X‐rays emitted by the irradiated foil. The location where the fluorescence signal inside the camera vanishes is used to extrapolate the location of the field boundary. Monte Carlo simulations were performed to determine the optimal composition and thickness of the foil. A prototype camera with a 1‐mm‐wide slit was built and tested in a clinical mammography and digital breast tomosynthesis (DBT) system.

**Results:**

The simulations estimated that a foil made of 25 µm of Molybdenum provided the maximum signal inside the camera for a 39 kVp beam. The boundary of the X‐ray fields in mammography and DBT views were experimentally measured. With the camera along the chest wall side, the measured field in multiple DBT views had a variability of only 0.4 ± 0.1 mm compared to mammography. A difference in the measured boundary position of 2.4 and –1.0 mm was observed when comparing to measurements with a fluorescent ruler and self‐developing film.

**Conclusion:**

The introduced technique provides a practical alternative method to detect the boundary of an X‐ray field. The method can be combined with other testing methods to assess the congruence of the X‐rays and light fields, and to determine if the X‐ray field extends beyond the detector more than permitted.

## 1 INTRODUCTION

One of the essential components of a clinical X‐ray imaging system is a beam‐limiting device that defines a rectangular field‐of‐view (FOV) on the detector plane. The beam‐limiting device is typically constructed with two pairs of orthogonal collimator plates. Some systems also have a mirror below the focal spot that reflects a light beam through the beam‐limiting device, providing a visible projection of the FOV that simplifies patient positioning before irradiation. To maximize the recorded anatomical information, the collimated X‐ray field is, in common, slightly larger than the rectangular image receptor. However, radiation emitted outside the detector delivers unnecessary radiation dose to the patient and produces unwanted scatter. A misalignment between the X‐rays and light fields can result in faulty images that need to be retaken. For these reasons, methods to evaluate the congruence between the X‐ray field, the light field, and the active area of the image receptor have been created.

In the particular case of mammography systems, regulatory agencies mandate strict limits on the collimation accuracy and prescribe corresponding quality control activities that need to be performed in devices used in clinical practice. In the United States, the Code of Federal Regulations^1^ mandates that film‐based mammographic beam‐limiting devices shall be provided with a means to limit the useful beam such that the X‐ray field at the plane of the image receptor does not extend beyond any edge of the image receptor by more than 2% of the source‐to‐image detector distance (SID). The Mammography Quality Standards Act (MQSA)^2^ regulations also require an X‐ray field/light field/image receptor/compression paddle alignment assessment as part of the annual quality control tests for all screen‐film systems (MQSA Sec. 900.12 [e][5][vii]). This regulation mandates that the mammographic beam must extend beyond the chest wall side of the image receptor but not by more than 2% of the SID. In addition, if the system has a light field it shall be aligned with the X‐ray field so that the sum of the misalignments in length and width does not exceed 2% of the SID. For full‐field digital mammography and digital breast tomosynthesis (DBT) devices, similar limits in the fields congruence are required by the applicable manufacturer quality control protocols or by the quality assurance program recommended by the American College of Radiology (ACR) digital mammography quality control manual.^3^ The ACR quality control manual leaves to the discretion of the medical physicist the choice of the most appropriate tool to test the collimation congruence, and lists as possible options: film, computed radiography plate, self‐developing film, or electronic radiation rulers. The European guidelines for quality assurance in breast cancer screening and diagnosis^4^ also recommend the use of film, computed radiography plates, or self‐developing film for the alignment of X‐ray field/image receptor test (Sections 2a.2.1.1.3 and 2b.2.1.1.3), and specify that the X‐ray field shall not extend more than 5 mm outside the detector.

Current test methods to assess the congruence between the X‐ray field and the detector assume that the source is static and perpendicular to the detector plane. These two assumptions do not hold in DBT projections. In this modality, the X‐ray field may extend substantially beyond the detector at large acquisition angles if the collimator aperture is fixed. There are no established methods to measure the X‐ray field in individual DBT views. In one of the few publications that have addressed this issue, Popova et al.^5^ used radiosensitive film and an electronic radiation ruler with customized acquisition software to measure the FOV in DBT views. The aim of Popova's work was to assess the performance of a dynamic collimation system implemented in GE Healthcare's SenoClaire DBT system,* which moves the collimator blades during the DBT sweep to keep the FOV close to the detector boundaries.

In this article, we propose a new method to estimate how far the X‐ray field extends beyond the detector active area. The method can be used in radiography and the DBT system. The main advantage is the use of the standard image detector instead of an external sensor or film. Example use in a clinical mammography and DBT system is presented.

## 2 MATERIALS AND METHODS

### 2.1 Slit camera measuring system

We propose to use the built‐in detector in a clinical imaging system to measure the distance to the outer limit of the X‐ray field beyond the detector by transforming the transmission image acquisition into an emission image acquisition. The emission signal consists of secondary radiation produced by an object blocking the primary X‐ray beam at a known height above the detector. A slit camera, a linear extension of a pinhole camera, is used to project the secondary radiation onto the detector, enabling the visualization of the field boundary in the acquired image.

When a thin, flat foil of material is positioned above and parallel to the detector plane, some of the primary X‐rays coming from the X‐ray source will be absorbed by photoelectric interactions with the foil atoms. After photoelectric absorption, the atoms are ionized in a high‐energy state and quickly absorb an electron and emit the excess energy in the form of Auger electrons or one or more fluorescent (or characteristic) X‐rays with specific energies. These secondary particles are emitted in random directions. Some other primary X‐rays will not interact with the foil, while others will change direction due to Compton or Rayleigh scattering interactions. A slit camera located between the foil and the detector projects the secondary radiation emitted in the foil onto the detector. The purpose of the projection process is to allow imaging of the boundary of the beam located beyond the detector inside the detector.

**FIGURE 1:**
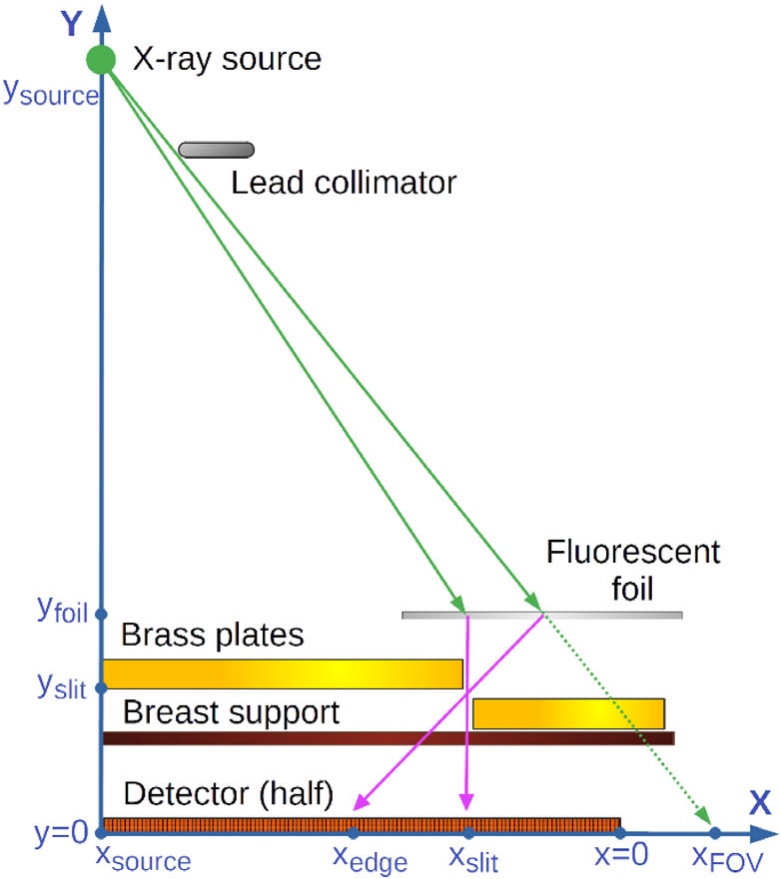
Diagram showing the operation of the slit camera in a mammography system (only half of the detector, from the center towards the right side, is shown). Two key primary X‐ray tracks, and their secondary fluorescence emission, are plotted with arrows. Thick brass plates, with a 1 mm gap in‐between, block substantially all the primary radiation so that only fluorescence generated in the thin foil and scatter can reach the detector. The boundary of the field‐of‐view (X_FOV_), which is located beyond the detector, is projected towards the detector through the slit camera aperture. The quantity of interest X_FOV_ can be estimated from the values of x_edge_ and x_slit_ measured in the image, and the known distances y_slit_, y_foil_, y_source,_ and y_source_

Figure 1 provides a simple diagram of the operation of the slit camera with the fluorescent foil in a mammography system. The diagram shows how to measure the size of the FOV at the right side of the detector. Analogous measurements with the foil and camera at different sides of the detector can measure the FOV in the left, chest wall, and nipple sides. In ideal conditions, the top plate of the camera should completely block the primary beam, and the image captured by the detector should show an uninterrupted signal from fluorescence radiation in the projection of the part of the foil inside FOV, and no signal beyond the FOV. The position of the abrupt transition between the two regions is determined by the position of the boundary of the FOV beyond the detector.

A slit camera, which captures much more light than a pinhole camera, can be used in this application because the edge we are imaging is a straight line. It is worth noting that in the quality control of X‐ray imaging systems, slit cameras have been used extensively to measure the size of the focal spot.^6^ We use the same projection principle, but imaging secondary radiation instead of the primary beam directly. Slit cameras, pinholes, and other general coded apertures have been used in other radiological imaging applications such as single‐photon emission computerized tomography,^7^ prompt gamma imaging in proton therapy,^8^ or small‐angle X‐ray scattering measurements.

**FIGURE 2:**
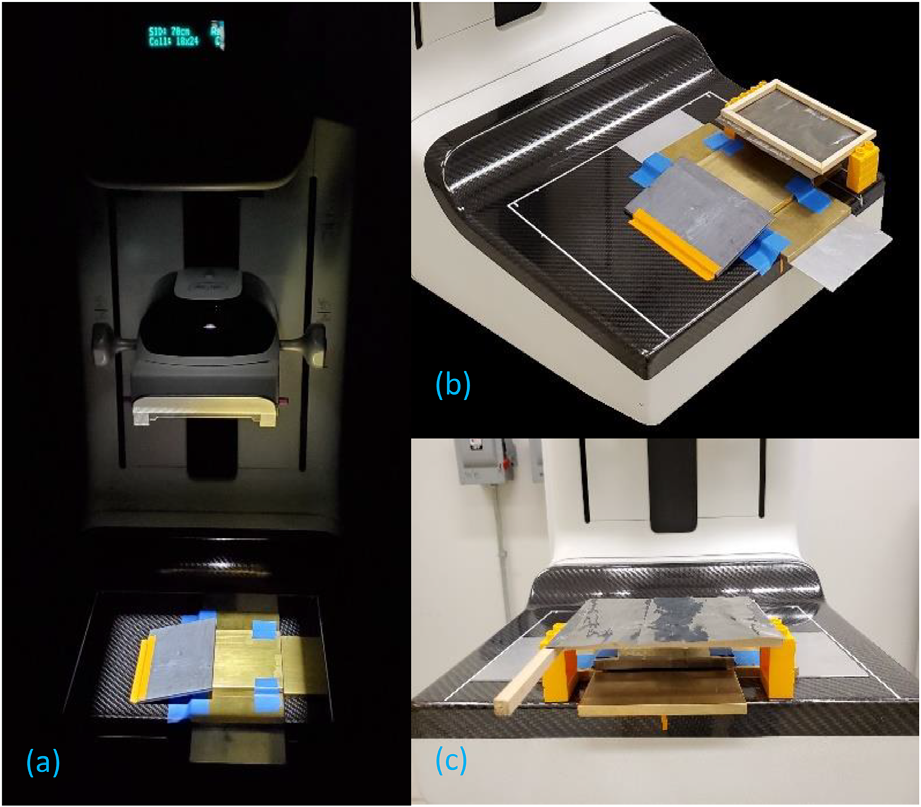
Prototype slit camera installed on a mammography/digital breast tomosynthesis (DBT) machine: (a) picture of the camera's brass plates blocking the primary beam and the light field presenting the region covered by the X‐ray field, (b) camera with a 25‐µm‐thick Molybdenum foil ready to measure the X‐ray field size at the right side of the detector, and (c) camera and foil set to measure the X‐ray field size at the chest wall side of the detector. The 1‐mm‐wide slit aperture is clearly visible

Figure 2 presents pictures of a prototype slit camera system installed on a Hologic Selenia Dimensions mammography and DBT system (Hologic Inc., Marlborough, MA). The camera was built using 6.35‐mm‐thick brass plates.† Brass alloy 360, with a nominal composition of 61.5% Copper (Cu), 35.5% Zinc (Zn), 3% Lead (Pb), and density 8.5 g/cm^3^, was chosen because it has a large attenuation coefficient for mammographic beams, and has the practical advantages of being low‐cost and easily machinable. The top of the camera was a 10 × 10 cm brass plate, suspended above the breast support by two 10 × 5 cm side brass plates and 1 mm Aluminum (Al) spacer plates. In this mammography system, the breast support is 2.5 mm thick and located 25.0 mm above the detector plane (the anti‐scatter grid can be inserted between the support and the detector, but it was not used in our experiments). The front of the camera was a 10 × 5 cm brass plate positioned approximately 0.5 mm ahead of the top plate. Thanks to the Al spacers, the lower part of the top plate was 1 mm above the upper part of the front plate. Therefore, the slit aperture size was 1 mm high, 0.5 mm wide, and 100 mm long. In practice, the effective size of the aperture seen by the X‐rays depends on the incidence angle. The back of the camera was covered with a large Pb plate to minimize the area of the detector irradiated by the primary beam. A 15 × 10 cm metal foil was framed to remain flat, and suspended on 38.5‐mm‐tall plastic blocks above the breast support, with the edge of the light beam delimiting the irradiation field aligned with the center of the frame. The slit was also aligned parallel to the light field edge and positioned approximately 30 mm away from the edge toward the center of the detector.

The distance from the side of the detector (i.e., the first or last column or row of pixels, which we define as the origin of coordinates) to the end of the X‐ray FOV beyond the detector (X_FOV_) can be calculated using Equation (1). This equation (which was deduced using the similarity of triangles in the diagram in Figure 1 uses the position of the edge inside the slit camera measured in the image profiles (x_edge_) and the known positions of the foil, slit, and X‐ray source.

(1) $$\begin{array}{ccc} & & X_{\mathrm{FOV}}\\ & & \;=\;\frac{x_{\mathrm{edge}}+\left(\frac{y_{\mathrm{foil}}}{y_{\mathrm{slit}}}\right)\times \left(x_{\mathrm{slit}}-x_{\mathrm{edge}}\right)-\left(\frac{y_{\mathrm{foil}}}{y_{\mathrm{source}}}\right)\times x_{\mathrm{source}}}{1-\left(\frac{y_{\mathrm{foil}}}{y_{\mathrm{source}}}\right)}\; \end{array}$$

The horizontal position of the source focal spot (x_source_) is near half the detector width when measuring the lateral side of the detector, and near 0 when measuring the chest wall side. The exact position in three dimensions of the focal spot in our experimental setting was measured with a geometric calibration phantom composed of ball bearings in known positions in two planes separated by 75.5 mm, as described by Li et al.^9^

Determining the exact position of the edge inside the camera (x_edge_) is challenging because the edge is expected to be blurred by the finite aperture of the slit, X‐rays transmission through the slit walls, the unwanted signal from scattering generated in the breast support, and inherent detector resolution limits. In addition, the boundary of the FOV is not sharp (penumbra) due to the finite size of the focal spot, transmission through the collimator edge, and the presence of off‐focal spot radiation. For reliable and repeatable measurements, the edge location was extrapolated from a linear interpolation fitted to the points in the steep intensity fall leading to the edge.

### 2.2 Fluorescent foil optimization

There are five main requirements on the fluorescent foil material used in the proposed slit camera: A high X‐rays attenuation to allow the use of a thin foil that emits radiation at a well‐defined plane, K‐edge energy low enough that a substantial fraction of the X‐rays emitted by a diagnostic imaging source can produce fluorescence, a large fluorescence yield after photoelectric effect, a K_alpha_ fluorescence energy sufficiently high to pass through the detector cover and provide a measurable signal, and the commercial availability of stable and solid foils of the material. In an alternative embodiment of the proposed method, a material that produces a large amount of scattering could be utilized, but this option was not studied in this work.

For the application of the proposed method in mammography, which is our first objective, Molybdenum (Mo) appeared as a natural option for the foil material, because this metal is used as a target material and filter in many commercial mammography systems. Since the Mo K‐edge is used to limit the maximum energy in clinical beams, its K_alpha_ emission, with an energy slightly below the K‐edge, should have an energy within the optimal detection range of mammography detectors, while being energetic enough to pass through the breast support and detector cover. Other elements with an atomic number right below Mo, such as Zirconium (Zr), are of interest because their lower K‐edge energy enables the generation of fluorescence from the X‐rays generated in a source using a Mo target and Mo filter. Table 1 provides the fluorescence parameters for Mo and other elements of interest as provided in the National Institute of Standards and Technology reference databases.^10^, ^11^

**TABLE 1:** Atomic relaxation (fluorescence) properties of some elements of interest for this work as provided in the NIST reference databases. The reported K‐edge corresponds to the binding energy of the atomic K shell of the corresponding element, and the yield corresponds to the total radiative yield of the K_alpha_ emission

**Element**	**Atomic number**	**Density [g/cm^3^]**	**K‐edge [eV]**	**K_alpha_ [eV]**	**Yield [%]**
Copper (Cu)	29	8.96	8 981	8 048	45.4
Zirconium (Zr)	40	6.51	17 996	15 775	73.4
Molybdenum (Mo)	42	10.22	20 000	17 479	76.7
Silver (Ag)	47	10.50	25 516	22 163	83.1
Tin (Sn)	50	7.31	29 200	25 271	86.0

Table 1 shows that Cu, a material commonly used in X‐ray imaging experiments, has a low fluorescence yield and characteristic energy that is too low to be reliably detected below the breast support plate of a mammography machine. Zr and Mo have high yields and optimal fluorescence energy for a mammography detector. In addition, thin foils of these materials are chemically stable and readily available for purchase from chemical product suppliers. The mean free path for photoelectric interactions in brass is 22.3 µm for Mo K_alpha_ fluorescence, 17.0 µm for Zr, and 42.8 µm for Ag. Therefore, the 6.35‐mm‐thick brass plates used in the prototype camera were about 150 times thicker than the mean free path of the most energetic X‐rays in the Ag‐filtered mammography beam. In principle, this attenuation was more than necessary to block the primary beam but was adequate for an initial prototype in which the total weight and ease of use are not relevant factors.

The optimal thickness of the foil material depends on the balance between two competing requirements: Thick foils maximize primary radiation absorption and the consequent secondary radiation generation, while thin foils maximize secondary radiation transmission towards the slit camera. The optimal foil thickness and composition were estimated using Monte Carlo simulations. The radiation transport simulation code PENetration and Energy LOss of Positrons and Electrons (PENELOPE) 2018^12^ with the modular penEasy^13^ main program was used to simulate the primary X‐rays, scatter, and fluorescence emission in a digital twin of the proposed device. PENELOPE uses state‐of‐the‐art models of fluorescence emission, including partial cross‐sections for the K shell and L, M, and N subshells of all neutral atoms.^14^ The geometry of the imaging system (schematically shown in Figure 1) can be easily modeled by intersecting volumes delimited by quadric surfaces,^15^ which is the standard geometry model used in PENELOPE. In our setting, the geometry requires only intersecting multiple planes, and a cylinder for the Pb collimator edge to produce a realistic penumbra at the limit of the field.

The simulated X‐ray beam was emitted from a cubic focal spot with 350 µm sides and reproduced the energy spectrum of a clinical source with a Tungsten (W) target, 39 kVp voltage, and 60 µm Silver (Ag) filtration (mean energy of 22.5 keV). The simulated photon and electron tracks were modeled down to 10 keV. For the foil thickness optimization, the scored quantity of interest was the total energy arriving at a 5 × 5 cm region of interest at the center of the camera. This quantity estimates the magnitude of the signal detected inside the camera from fluorescence X‐rays generated in the foil. The simulation was repeated for a range of thicknesses, and for both Zr and Mo foils. Recognizing the fact that the presented method can be used in radiographic applications other than mammography, we repeated the simulations with an X‐ray energy spectrum of 100 kVp and using Mo and Sn foils with up to 500 µm thickness.

The penEasy imaging detector tally was also used to simulate mammographic images of the slit camera device. The simulations allowed us to study the transmission of the primary beam through the top plate and the corners of the slit, study the contribution of fluorescence, Compton, and Rayleigh radiation generated in the foil and in the breast support to the signal detected inside the camera, and estimate the sensitivity of the device to measure small changes in the field size.

### 2.3 Validation with external sensors

The proposed methodology to measure the size of a radiographic field must be compared to established, commonly used methods to determine if the new process provides acceptable results. As an initial step in the validation process, we measured the field size in our mammography system using two commercial products: An RTI Nova optically fluorescent ruler set (RTI Group AB. & Inc., Mölndal, Sweden), and Gafchromic XR self‐developing film (Ashland Global Holdings Inc., Wilmington, DE). The external sensors were positioned across the chest side of the detector, and the position of the edge of the light field was marked with a metal indicator on the ruler, and with a pen line on the film. A mammographic exposure was made with the same parameters used with the slit assembly experiments. The room was kept in the dark, and a cellphone camera recorded the optical fluorescence emitted by the ruler.

## 3 RESULTS

**FIGURE 3:**
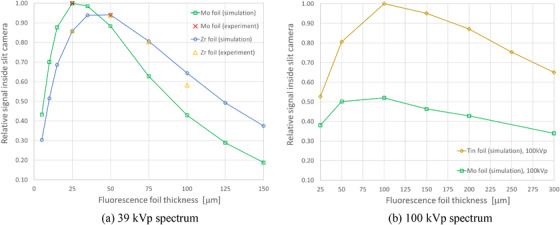
Optimization of the fluorescence foil material and thickness using Monte Carlo simulations with the PENELOPE code, and corresponding experimental results, for (a) 39 kVp and (b) 100 kVp spectra. The reported signal was the average pixel value inside the slit camera. The simulated results were normalized by the largest reported signal. The two sets of experimental results were separately normalized to match the simulated signal with 25 µm of material

The results of the PENELOPE simulations estimating the signal detected inside the slit camera for a range of Mo and Zr foil thicknesses are provided in Figure 3. The results show that the maximum signal for the 39 kVp spectrum was obtained with 25 µm of Mo. The maximum signal with Zr, only 6% smaller than the Mo maximum, was obtained with a thickness of 50 µm. The optimization process was not further refined for other intermediate thicknesses near the peaks because there are not commercially available foils at arbitrary thicknesses. For the 100 kVp spectrum, 100 µm of Sn foil gave the largest signal, nearly doubling the signal from the same thickness of Mo.

Figure 3 also presents experimental measurements with the prototype device in the Hologic Selenia Dimensions mammography machine using multiple layers of 25 µm foils. To compare with the simulations, the experimental results were normalized to match the simulated signal at 25 µm for the corresponding material.

**FIGURE 4:**
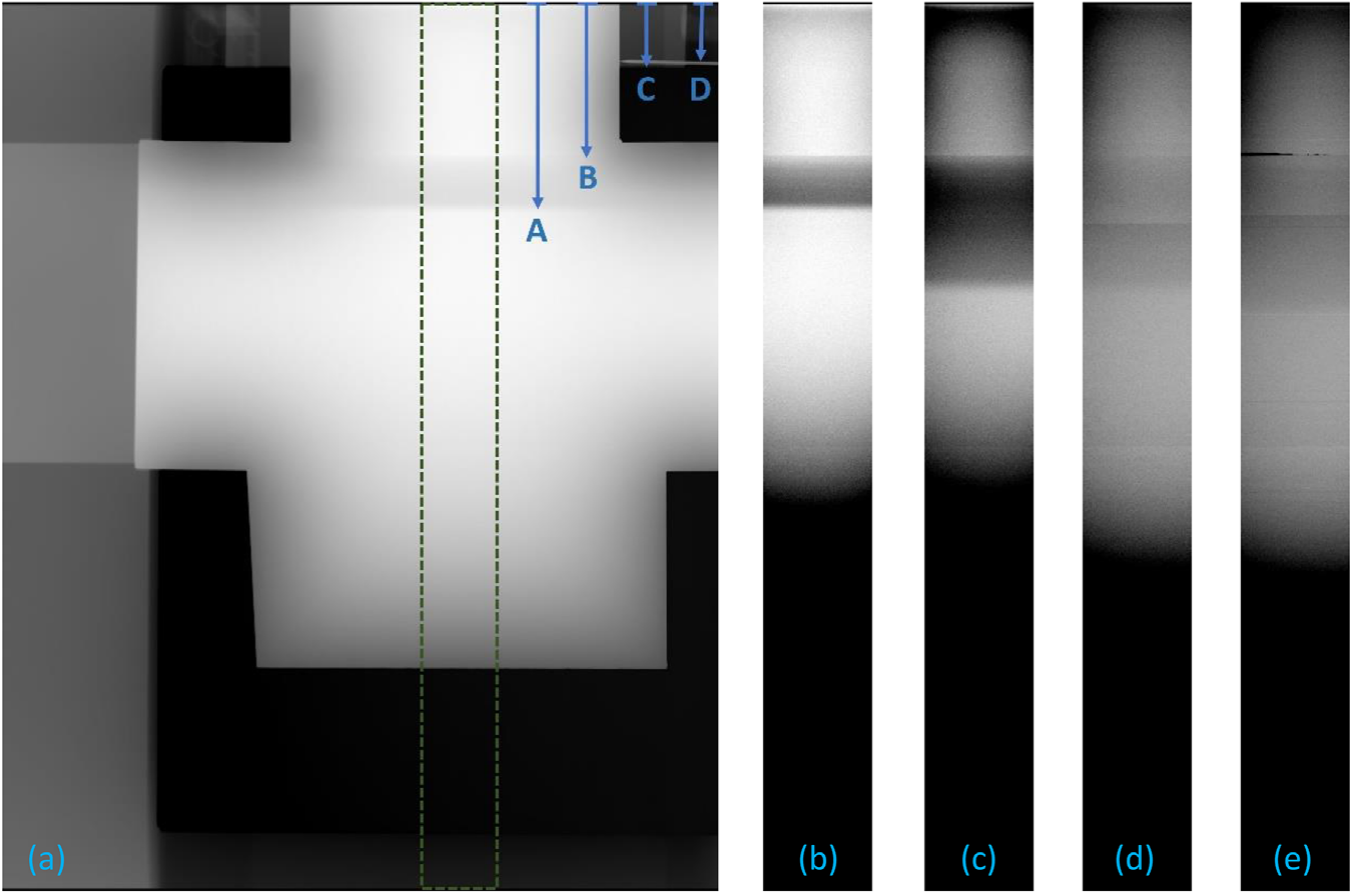
(a) Mammographic image (in log scale) of the slit camera installed at the right side of the detector (visualized at the top of the image) with the X‐ray field collimated to 18 × 24 cm (smaller than the full detector). The following distances from the right side of the detector are marked with the blue arrows: A–edge of the fluorescence signal projected inside the camera (x_edge_), B–slit aperture position (x_slit_), C–end of collimated X‐ray field‐of‐view (X_FOV_), D–needle aligned with the light field (x_light_). The dashed rectangle indicates the 500‐pixel‐wide region of interest (ROI) shown in the sub‐figures for different acquisitions: (b) Mammography with 18 × 24 cm field, (c) Mammography with 24 × 29 cm field (default full field), (d) central digital breast tomosynthesis (DBT) projection at 0°, and (e) last DBT projection at +7.5°. The DBT projections were scaled 2 × 2 for display purposes to compensate for the default pixel binning in DBT acquisitions. All the ROI figures are presented in linear scale and with a common grayscale (level 130, window 100)

An experimental raw projection image (in “for processing” mode) of the prototype slit camera acquired with the clinical system is shown in Figure 4a. Figures 4b,c show regions of interest (ROI) of the signal acquired with the X‐ray field collimated to 18 × 24 cm and 24 × 29 cm (full field), respectively. The two field sizes were defined using the source built‐in Pb collimator. The former field was smaller than the detector and its limits can be seen in the radiographic image (except in the chest wall side); the latter full‐field covered the entire detector. Figures 4d,e show the signal in the DBT projections at 0° (equivalent to a mammogram orientation) and the maximum angulation of 7.5°.‡ For the FOV smaller than the detector shown in Figure 4a, the distance from the top of the image to the X‐ray field boundary, C, was 21.1 mm, while the distance to the boundary of the light field marked by a radiopaque needle, D, was 19.0 mm.

The acquired images had 3328 × 4096, 70 µm pixels in mammography, and 1664 × 2048, 140 µm in DBT (2 × 2 binning). The manual imaging settings used for the mammography acquisitions were 39 kVp and 450 mAs, which were the maximum values allowed by the system. A 50‐µm‐thick Ag filter was used to produce a harder beam (i.e., containing more high‐energy X‐rays above the Mo K‐edge) than the default Rhodium filter. For the DBT projections, 39 kVp and 125 mAs were used. The system automatically used a 700‐µm‐thick Al filter in DBT mode, and therefore the beam contained even more high‐energy X‐rays than with the Ag filter because Al does not have an absorption edge in the energy range of interest. The built‐in anti‐scatter‐grid would absorb the fluorescence radiation and interfere with the slit camera operation, and therefore it was retracted for all acquisitions.

**FIGURE 5:**
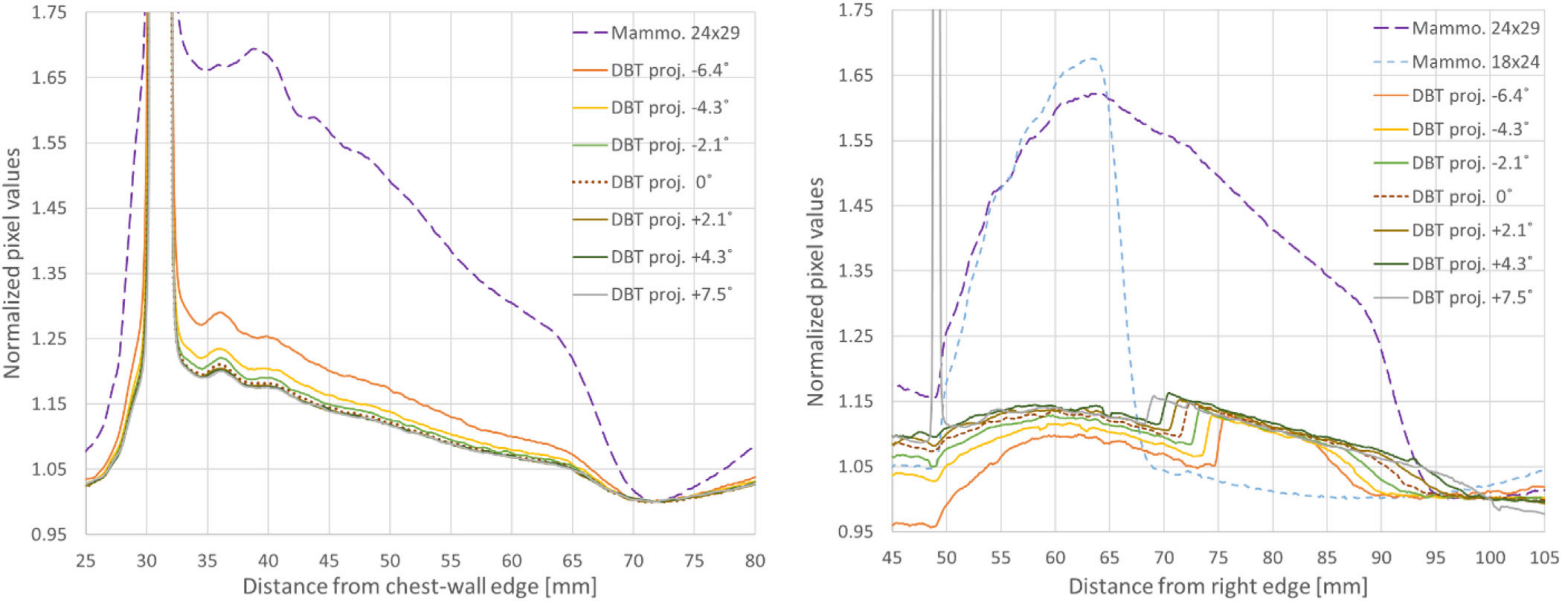
Integrated line profiles inside the slit camera for mammography and digital breast tomosynthesis (DBT) projections: (left) slit camera aligned with the chest wall side of the detector, and (right) slit camera aligned with the right side of the detector. The DBT and mammography acquisition with the full field size of 24 × 29 cm were acquired consecutively in combo mode

Figure 5 summarizes the information in the ROI images in Figure 4 in the form of line intensity profiles. The profiles were generated by integrating the width of the ROIs and then convolving with a 5‐pixel‐wide mean filter for noise reduction. The integration assumes that the slit was accurately aligned with the side of the detector (poor alignment would result in blurring of the fluorescence edge). Each profile was normalized to its minimum intensity inside the camera, to compensate for differences in exposure and incidence angle in each projection. The line profiles with the slit camera aligned with the chest wall side of the detector (not shown in Figure 4) are also presented. In the chest wall configuration, the plane of rotation of the source in DBT was parallel to the slit and, consequently, the field size is expected to remain equal to that in the mammogram at all angles.

**FIGURE 6:**
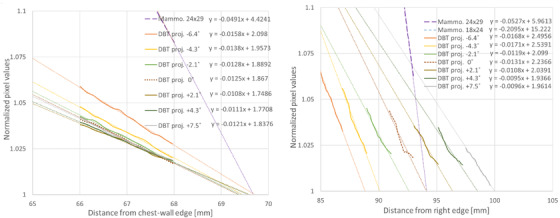
Fitting linear trendlines to the linear section leading to the slit camera edges in Figure 5. The fitting equations are shown in the legend (correlation coefficients (*R* ^2^) varied between 0.93 and 0.99). The extrapolation of the trendlines to the minimum value of 1 (the normalization point) was assumed to be the edge position (x_edge_)

To determine the position of the fluorescence signal edge inside the slit camera, linear fits were performed to the linear section of the profiles leading to the edges in Figure 5. The fittings are shown in Figure 6. The edge position (x_edge_) was found by solving the fitting equations for y = 1. Table 2 presents the measured edge positions and the corresponding FOV positions calculated with Equation (1). All distances are reported with respect to the nearest side of the detector, with negative distances representing a position outside of the detector and positive distances inside the detector. The following geometric parameters were used in the equation: y_foil _= 63.5 mm; y_slit _= 31.35 mm; x_slit _= 50.0 mm (right side), 33.0 mm (chest wall side); y_source _= 661.2 mm; and x_source _= 133.7 mm (right side), 23.6 mm (chest wall side). These parameters were measured on the physical prototype with a caliper or processing the radiographic image.

**TABLE 2:** This table reports the positions of the edge of the fluorescence signal inside the slit camera (x_edge_) measured in the line profiles from the experimental images, the corresponding positions of the end of the X‐ray field‐of‐view (X_FOV_) calculated with Equation (1), and the distance between each field and the full‐field mammography field, for different views and two sides of the detector. Negative distances indicate that the X‐ray field boundary is outside the detector

**Camera orientation**	**View**	**x_edge_ (mm)**	**X_FOV_ (mm)**	**Difference to mammo.(mm)**
Chest wall side	Mammo., 24 × 29	69.74	−7.68	0.00
	DBT proj., −6.4°	69.49	−7.41	0.28
	DBT proj., −4.3°	69.37	−7.27	0.42
	DBT proj., −2.1°	69.47	−7.38	0.30
	DBT proj., 0°	69.36	−7.25	0.43
	DBT proj., +2.1°	69.31	−7.20	0.48
	DBT proj., +4.3°	69.44	−7.35	0.34
	DBT proj., +7.5°	69.22	−7.10	0.58
Right side	Mammo., 24 × 29	94.14	−8.97	0.00
	Mammo., 18 × 24	67.89	20.82	29.79
	DBT proj., −6.4°	89.02	−3.16	5.81
	DBT proj., −4.3°	90.01	−4.28	4.69
	DBT proj., −2.1°	92.35	−6.94	2.03
	DBT proj., 0°	94.40	−9.26	−0.29
	DBT proj., +2.1°	96.21	−11.32	−2.35
	DBT proj., +4.3°	98.59	−14.02	−5.05
	DBT proj., +7.5°	100.15	−15.78	−6.81

Abbreviations: DBT, digital breast tomosynthesis; mammo., mammography; proj., projections.

**FIGURE 7:**
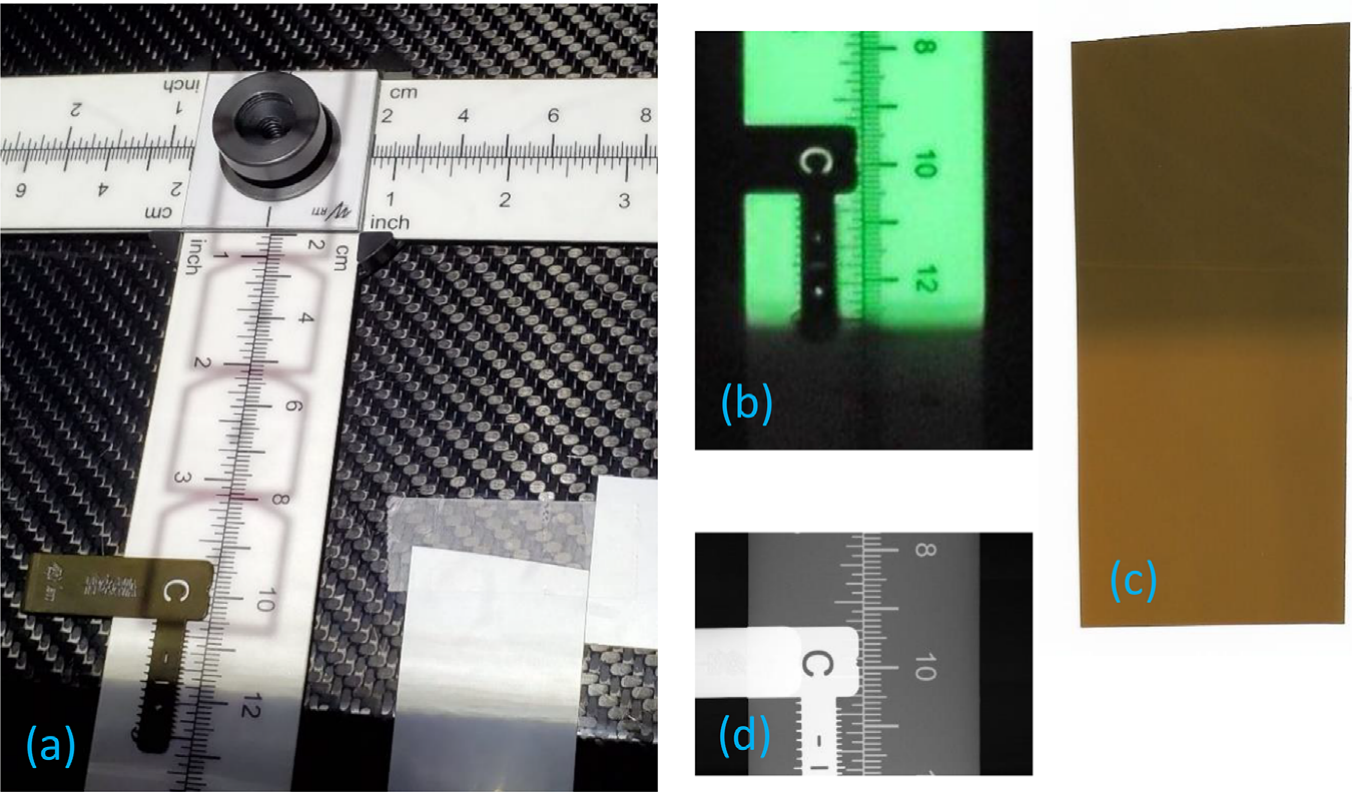
Measurement of the position of the X‐ray field beyond the chest side of the mammography detector using an RTI Nova optically fluorescent ruler and a Gafchromic XR film strip: (a) Picture of the external sensors on the breast support, (b) video frame showing the ruler fluorescence during the irradiation, (c) flatbed scan of the self‐developed film after irradiation (a faint white horizontal line marks the position of the light field edge), and (d) portion of the X‐ray projection image showing the radiopaque light field position marker near the chest edge of the detector

Finally, Figure 7 shows the measurement of the size of the X‐ray field in the chest side of the mammography detector using two external sensors. The boundary of the irradiation field and the markers of the light field edge were clearly visible in a frame of the video recording of the optically fluorescent ruler (Figure 7b), and also in the self‐developed film (Figure 7c). The distance between the light and X‐ray fields was measured to be 11.2 mm in the fluorescent ruler, and 7.9 mm in the film. These measurements were taken on top of the breast support and had to be scaled to the detector plane 25.0 mm below. The edge position measurements had a substantial uncertainty due to the penumbra effect that, expectedly, prevented having sharp edges in both the light and X‐ray fields. The metallic marker indicating the position of the light field edge on the ruler was visible in the X‐rays projection image (Figure 7d). The distance between this light field marker and the end of the detector was 1.5 mm, as measured in the image (detector plane) knowing the pixel size of 70 µm. Projecting the distances measured in the ruler and film to the detector plane and subtracting the distance to the edge of the detector measured on the radiograph, we estimated that the X‐ray field extended 10.1 mm beyond the detector in the fluorescent ruler measurement, and 6.7 mm in the film measurement. These distances can be compared to the reported distance of 7.68 mm in the first row of Table 2 for the slit camera experiment.

## 4 DISCUSSION

The presented methodology successfully measured the distance to the X‐ray field boundary in a clinical system. The measured fluorescence signal below the camera was extremely small. The pixel values output by the mammography system ranged between 50 (detector offset) and 16 383 (14‐bit) analog‐to‐digital units (ADU). The pixels in the part of the camera irradiated by fluorescence had values of the order of 150 ADU, while the pixels under the camera without fluorescence signal had values of the order of 100 ADU, due to unwanted scatter generated outside the camera and transmission through the camera top plate. Therefore, the fluorescence signal had a very low magnitude of the order of 50 ADU above the background. Fortunately, it was possible to reliably measure this low signal thanks to the integration of many pixels into a line profile, as seen in Figure 5. A longer slit could be used to integrate more pixels in the line profiles. Multiple acquisitions or larger mAs could also be used to reduce the signal‐to‐noise ratio.

The X‐ray field positions reported in the Table 2 were within the expected range. The measurement in the chest wall side of the detector for the DBT and mammography projections (field size 24 × 29 cm) had an excellent agreement, with an average difference between the DBT views and mammography of only 0.4 ± 0.1 mm. On the right side, the agreement between the mammogram and the DBT view at 0° was 0.29 mm. These results suggest that the measurement has good repeatability and potentially a sub‐millimetric precision. The distance measured for the small mammography field of 18 × 24 cm was 20.82 mm, which is in good agreement with the value of 21.1 mm measured directly on the projection image. Note that this smaller field size collimated only the lateral and anterior sides, and the field still extended beyond the chest side of the detector and could not be directly measured with the built‐in detector.

An interesting artifact can be seen in the right side DBT profiles in Figure 5. A sudden increase in the profile intensity can be clearly seen around 70 mm. The intensity increase happens at the projection of the location where the Mo foil finishes, indicating that there is some transmission of the beam through the top of the camera in the absence of foil. This feature is not visible in the mammography profiles because the Ag filter in the source effectively eliminates the higher energy rays that can penetrate the thick brass plate, unlike the Al filter used in DBT. In the same figure, a large intensity is seen around 50 mm for the DBT projection at +7.5°. This is caused by the primary beam entering directly through the slit at that angulation.

The distance to the X‐ray field boundary beyond the chest side of the detector measured by the two external sensors had a discrepancy of −1.0 mm for the film and 2.4 mm for the fluorescent ruler when compared to the new method. An error of the order of magnitude of a millimeter is comparable to the order of the uncertainty in the position of the edges caused by the penumbra (which might also explain some of the discrepancies between the ruler and the film). This provides initial experimental evidence that the new method is comparable to the two established methods using external sensors, even though refinements of the new device and methods are necessary to improve the accuracy. More validation activities (and done in the four sides of the detector to understand the impact of the heel effect, for example) are needed to completely characterize the performance of the new method.

## 5 CONCLUSIONS

This article introduced a new approach to measuring the congruence between the X‐ray field, the light field, and the image detector using X‐rays fluorescence and a slit camera. The main advantage of the method is the use of the built‐in detector in the system to measure X‐ray fields that extend beyond its physical limits, avoiding the need to use external radiation sensors for initial beam alignment and quality control. An optimization process based on Monte Carlo simulations determined that a 25‐µm‐thick Mo foil maximized the signal detected inside the slit camera for a 39 kVp mammography spectrum. For radiographic applications using X‐rays closer to a 100 kVp spectrum, 100 µm Sn foil was found to be a good option. A prototype of the described slit camera system was built and used to measure the X‐ray field size in combined mammography and DBT acquisition with a clinical imaging system. The camera was sensitive enough to detect the field size in each individual DBT projection. The measurements have good repeatability, with only 0.4 ± 0.1 mm variation in the measured field size in the chest wall side of the detector for the DBT views compared to the reference mammography acquisition. An initial experimental validation showed a discrepancy of less than 2.5 mm between the new method and established methods with external sensors.

## CONFLICT OF INTEREST

The author declares to have no conflict of interest.

## Data Availability

The data that support the findings of this study are available from the author upon reasonable request.

## Footnotes

* The mention of commercial products herein is not to be construed as either an actual or implied endorsement of such products by the Department of Health and Human Services.

† The brass plates of the appropriate size were sourced from https://www.cut2sizemetals.com. The metal foils were purchased from Alfa Aesar, part of Thermo Fisher Scientific (Tewksbury, MA).

‡ The individual DBT projection images were obtained from the secondary capture image file thanks to a proprietary conversion software provided by Hologic Inc.
